# Wound infection caused by *Neisseria zoodegmatis*, a zoonotic pathogen: a case report

**DOI:** 10.1099/acmi.0.000196

**Published:** 2021-02-10

**Authors:** John Merlino, Timothy Gray, Rohan Beresford, Sai Rupa Baskar, Thomas Gottlieb, Jacob Birdsall

**Affiliations:** ^1^​ Department of Microbiology and Infectious Diseases, Concord Hospital, NSW Health Pathology, Concord, NSW, Australia; ^2^​ Department of Medicine and Health, University of Sydney, Sydney, NSW, Australia

**Keywords:** animal bites, antibiotic susceptibility, Etest, MALDI-TOF MS, *Neisseria animaloris*, *Neisseria zoodegmatis*, *Pasteurella multocida*

## Abstract

The isolation of *Neisseria zoodegmatis* from a 63-year-old female presenting to the emergency department following a cat bite injury to her right hand is described in this report. *N. zoodegmatis *, also known as Centers for Disease Control (CDC) group EF-4b, is considered to be a zoonotic pathogen, and is usually associated with dog or cat bites. Despite the potential of this organism to cause serious soft tissue infections, it can be overlooked in routine clinical laboratories due to its slow growth characteristics and when the history of animal bite is not provided to the laboratory. This case highlights the importance of appropriate clinical history provision to the microbiology laboratory to help provide important information about potential pathogens and allow microbiologists to optimize culture and identification methods. The introduction of tools such as matrix-assisted laser desorption/ionization time-of-flight mass spectrometry (MALDI-TOF MS) into clinical laboratories allows identification and the interpretation of results to be performed within a few minutes of isolation on proper culture media, as opposed to traditional methods, whose slowness may be problematic, as shown in this case report.

## Introduction

Bacterial skin infections are common presentations to both general practice and hospital emergency departments. Without a proper clinical history, most clinical laboratories tend to culture for *Staphylococcus aureus* and/or beta-haemolytic streptococci, which account for the majority of pathogens associated with common skin and soft tissue infections [1]. Skin infections associated with unusual exposures and other clinical scenarios are sometimes neglected without a proper clinical history. Cultures from unusual exposures may require extended incubation and pathogens may be missed if not directly sought, which may lead to further complications from inappropriate antimicrobial therapy [2]. In this case report we describe the isolation, identification and antimicrobial susceptibility testing of *Neisseria zoodegamtis*, also known as Centers for Disease Control (CDC) group EF-4b, a pathogen of wound infections from cat bites that can be dismissed or misidentified. This is important, as delays in diagnosis and inappropriate antibiotic treatment can contribute to chronic wounds and poor patient outcomes [3].

## Case report

A 63-year-old female presented to the emergency department in a large urban hospital following a cat bite injury (from her own pet cat) to her right hand several hours previously. She was right-hand dominant and had no significant past medical history of note, including no regular medications and no known drug allergies. On presentation, she was afebrile and systemically well. There was soft tissue swelling of the thenar eminence of the right hand with several small punctures noted. She received routine aluminium hydroxide-adsorbed diphtheria and tetanus toxoids (ADT) vaccine and was empirically given oral amoxicillin/clavulanate 875/125 mg within 4 h of the injury. She was discharged home overnight, before a planned admission under the plastic surgery team the following morning. She was commenced on intravenous amoxicillin/clavulanate 1000/200 mg 8 hourly and underwent operative debridement within 24 h of the initial injury, at which time the intraoperative findings included frank pus over the thenar muscles.

An intraoperative specimen was collected for microbiology and the clinical history of cat bite was communicated to the laboratory. Small colonies were seen after 24–48 h and were identified as *Neisseria* species, with discrepant results between matrix-assisted laser desorption/ionization time-of-flight mass spectrometry (MALDI-TOF MS) and VITEK 2 (bioMérieux, Australia), which reported *Neisseria zoodegmatis* and *Neisseria weaveri *, respectively. The MALDI-TOF MS gave an identification of *N. zoodegmatis* with a score of 2.30, whilst the VITEK 2 XL with software 9.2 using the NH identification card reference 21 346 (bioMérieux, Australia) gave an identification as *N. weaveri* with an excellent identification of 99 % probability. The discrepancy was resolved by PCR using 16 s rDNA with 100 % match to *N. zoodegmatis* with 38–445 base pairs analysed using primers described by Flendrie *et al.* (forward: 5′-CCTAACACATGCAAGTCGARCG-3′; reverse: 5′CGTATTACCGCGGCTGCT-3′) [4]. Susceptibility testing was performed using Etests (bioMérieux, Australia), confirming low minimum inhibitory concentration (MIC) values to amoxicillin/clavulanate (Table 1) . Whilst an inpatient, she received 4 days of intravenous amoxicillin/clavulanate 1000/200 mg 8 hourly. She remained systemically well during her admission and there were no complications. She was discharged on day 5 of admission on oral amoxicillin/clavulanate 875/125 mg twice daily to complete a 10-day course [5].

**Table 1.** Results of antibiotic minimum inhibitory concentration (MIC) tests for the *N. zoodegmatis* isolate performed using Etest strips

Antibiotics	PG	AM	MICs mg l^−1^	AUG	CI
Breakpoints*	S <=0.25 R >2	S <=2, R >8		S <=2, R >8	S <=0.5, R >1
* N. zoodegmatis *	0.5	0.25		0.5	0.016

*Swedish Reference Group for Antibiotics as described previously [3].

PG, penicillin G; AM, ampicillin; AUG, amoxicillin/clavulanate; CI, ciprofloxacin.

## Discussion

Group EF-4 bacteria comprise two organisms, *Neisseria animaloris* (EF-4a) and *Neisseria zoodegmatis* (EF-4b) [6]. These two organisms are Gram-negative, coccoid and bacilli-like, and are considered to be commensals of the oral cavity in dogs, cats and rodents, like *Pasteurella* species. These organisms have been associated with severe soft tissue infections following animal bites, and other infections, including osteomyelitis and septicaemia [2, 7]. Both are slow-growing organisms that may require up to 48 h incubation on both blood and chocolate solid agar media in ambient air, but do not grow on MacConkey agar. Colony growth may appear as pinpoint colonies at 24 h on horse blood and chocolate agars, whilst further incubation to 48 h may show larger round yellow-white pigmented colonies at 5 % carbon dioxide. Biochemically, both *N. animaloris* and *N. zoodegmatis* are oxidase-positive, and may be misidentified as *Pasteurella multocida *; however, unlike most *Pasteurella *, both are indole-negative [8](Table 2)

**Table 2.** Phenotypic key characteristics differentiating *N. zoodegmatis, N. animaloris*, *N. weaveri* and *P. multocida
*

Species	Catalase	Oxidase	Indole	MacConkey	Glucose	Nitrate reduction	Nitrite reduction	Gas produced from nitrite	Arginine dihydrolase
* N. zoodegmatis *	Pos	Pos	Neg	No growth	Pos	Pos	Pos	Neg	Neg
* N. animaloris *	Pos	Pos	Neg	No growth	Pos	Pos	Pos	Pos	Pos
* N. weaveri *	Pos	Pos	Neg	No growth	Neg	Neg	Pos	Neg	Neg
* P. multocida *	Pos	Pos	Pos	No growth	Pos	Pos	Pos	Neg	Neg

Pos, positive reaction; Neg, negative reaction.

This case illustrates the importance of detailed clinical history taking and communicating relevant information to the microbiologist when a specimen is taken from a patient, and why it is important to have a good understanding of the clinical manifestations of infections due to the potential pathogens involved. In medical laboratories the clinical significance of *N. zoodegmatis* can easily be underestimated without a sufficient clinical history, while it can also easily be misidentified.

## References

[1] Abrahamian FM, Goldstein EJC (2011). Microbiology of animal bite wound infections. Clin Microbiol Rev.

[2] Oehler RL, Velez AP, Mizrachi M, Lamarche J, Gompf S (2009). Bite-related and septic syndromes caused by cats and dogs. Lancet Infect Dis.

[3] Heydecke A, Andersson B, Holmdahl T, Å M (2013). Human wound infections caused by *Neisseria animaloris* and *Neisseria zoodegmatis*, former CDC Group EF4a and EF-4b. Infect Ecol Epidemiol.

[4] Flendrie M, Jeurissen M, Franssen M, Kwa D, Klaassen C (2011). Septic arthritis caused by *Legionella dumoffi* in a patient with systemic lupus erythematosus-like disease. J Clin Microbiol.

[5] Broom J, Woods ML (2006). Management of bite injuries. Aust Prescr.

[6] Vandamme P, Holmes B, Bercovier H, Coenye T (2006). Classification of centers for disease control group eugonic fermenter (EF)-4a and EF-4b as *Neisseria animaloris* sp. nov. and *Neisseria zoodegmatis sp.* nov., respectively. Int J Syst Evol Microbiol.

[7] Andersen BM, Steigerwalt AG, O'Connor SP, Hollis DG, Weyant RS (1993). *Neisseria weaveri sp.* nov., formerly CDC group M-5, a Gram-negative bacterium associated with dog bite wounds. J Clin Microbiol.

[8] Oberhofer TR (1981). Characteristics and biotypes of *Pasteurella multocida* isolated from humans. J Clin Microbiol.

